# Diagnostic Performance of Whole-Body PET/MRI for Detecting Malignancies in Cancer Patients: A Meta-Analysis

**DOI:** 10.1371/journal.pone.0154497

**Published:** 2016-04-28

**Authors:** Guohua Shen, Shuang Hu, Bin Liu, Anren Kuang

**Affiliations:** Department of Nuclear Medicine, West China Hospital, Sichuan University, No. 37 Guoxue Alley, Chengdu, Sichuan, 610041, People’s Republic of China; North Shore Long Island Jewish Health System, UNITED STATES

## Abstract

**Background:**

As an evolving imaging modality, PET/MRI is preliminarily applied in clinical practice. The aim of this study was to assess the diagnostic performance of PET/MRI for tumor staging in patients with various types of cancer.

**Methods:**

Relevant articles about PET/MRI for cancer staging were systematically searched in PubMed, EMBASE, EBSCO and the Cochrane Library. Two researchers independently selected studies, extracted data and assessed the methodological quality using the QUADAS tool. The pooled sensitivity, specificity, diagnostic odds ratio (DOR), positive likelihood ratio (PLR), and negative likelihood ratio (NLR) were calculated per patient and per lesion. The summary receiver-operating characteristic (SROC) curves were also constructed, and the area under the curve (AUC) and Q* estimates were obtained.

**Results:**

A total of 38 studies that involved 753 patients and 4234 lesions met the inclusion criteria. On a per-patient level, the pooled sensitivity and specificity with 95% confidence intervals (CIs) were 0.93 (0.90–0.95) and 0.92 (0.89–0.95), respectively. On a per-lesion level, the corresponding estimates were 0.90 (0.88–0.92) and 0.95 (0.94–0.96), respectively. The pooled PLR, NLR and DOR estimates were 6.67 (4.83–9.19), 0.12 (0.07–0.21) and 75.08 (42.10–133.91) per patient and 10.91 (6.79–17.54), 0.13 (0.08–0.19) and 102.53 (59.74–175.97) per lesion, respectively.

**Conclusion:**

According to our results, PET/MRI has excellent diagnostic potential for the overall detection of malignancies in cancer patients. Large, multicenter and prospective studies with standard scanning protocols are required to evaluate the diagnostic value of PET/MRI for individual cancer types.

## Introduction

Cancer continues to be a major public health problem in the United States and many other parts of the world, and one in 4 deaths in the United States is due to cancer [1]. Knowing the exact tumor stage is essential for selecting the appropriate therapeutic strategies to provide the best available care and best prognosis for the patient.

Conventional imaging procedures, such as chest radiography, CT, and ultrasonography, are commonly used to detect malignant lesions and assess tumor staging. However, based only on the morphologic criteria, it is difficult to identify small lesions and to distinguish potential metastatic lesions from benign findings [2]. The integrated ^18^F-fluorodeoxyglucose positron emission tomography (FDG PET)/computed tomography (CT), which combines morphological and functional information, is helpful in tumor staging and is currently a major diagnostic tool in oncology [3,4]. Because it provides a higher level of accuracy in TNM staging than does either PET or CT alone, PET/CT is considered indispensable [5]. Magnetic resonance imaging (MRI), which has excellent soft-tissue contrast compared to CT, can improve tumor detection and delineation in body regions with difficult anatomy, such as the head and neck areas and gynecological regions. Integrated PET/MRI, which combines the excellent anatomical resolution and high soft-tissue contrast of MRI with the highly sensitive evaluation of metabolism and molecular processes of PET, has recently been applied in clinical practice as a new multimodality imaging [6,7]. Furthermore, functional MRI sequences, such as diffusion-weighted imaging (DWI) and other multiparametric sequences, can be added to the scanning protocol, which might enhance its diagnostic performance and predictive value [8,9]. Several published studies have shown the feasibility and efficacy of PET/MRI in tumor staging in various cancers [9–14]. Karsten et al have recently demonstrated the higher lesion conspicuity and diagnostic confidence of PET/MRI compared to PET/CT for the depiction and characterization of liver lesions [15]. PET/MRI is also a valuable technique for assessing primary tumor and nodal staging in patients with endometrial cancer as well as in patients with head and neck cancers [11,16]. However, these studies had relatively small sample sizes and limited power for an individual study.

In this study, we performed a meta-analysis to assess the diagnostic performance of PET/MRI in tumor staging systematically.

## Materials and Methods

### Search strategy and study selection

We searched for studies evaluating PET/MRI for tumor TNM staging in patients with various cancers, and relevant studies were identified with a comprehensive search of MEDLINE, EMBASE, EBCSO and the Cochrane Library from January 1, 2000 to October 1, 2015. The search strategy was based on the combination of (PET-MRI OR PET/MRI OR MRI-PET OR MRI/PET OR positron emission tomography/magnetic resonance imaging OR positron emission tomography-magnetic resonance imaging) AND (neoplasm OR cancer OR carcinoma) AND (staging OR diagnosis). References in the included studies were screened for additional studies.

Regarding the study selection, the inclusion criteria were as follows: (a) PET/MRI was used as a diagnostic tool for TNM staging in cancer patients; (b) there were sufficient data labeled as true-positive (TP), false-positive (FP), false-negative (FN) and true-negative (TN) results; (c) the data analysis was performed at either the patient level or the lesion level or both, and the minimal sample size was 10; (d) histopathologic results and/or clinical and imaging follow-up were used as the reference standard. We excluded reviews; meeting abstracts; letters; and case reports without absolute numbers of TP, FP, FN and TN estimates.

### Data extraction and quality assessment

Two investigators independently extracted data from the included studies, and discrepancies were resolved by discussion. For each study, we collected information on author names; publication year; origin country; patient characteristics (number of eligible patients/lesions, gender, year); cancer type (head and neck, breast, lung, prostate, or others); study design (prospective or retrospective); scanning modality; and reference standard. We also recorded whether the interpretation of the PET/MRI was blinded to the reference standard. In addition, for each study, the numbers of TP, FP, FN and TN findings were recorded to perform accuracy analyses.

The methodology of the included studies was evaluated by the quality assessment tool for diagnostic accuracy studies (QUADAS) [17]. This tool consists of 14 items: patient spectrum (item 1), reporting of selection criteria (item 2), appropriate reference standard (item 3), absence of disease progression bias (item 4), absence of partial verification bias (item 5), absence of differential verification bias (item 6), absence of incorporation bias (item 7), description of test execution details (item 8), description of reference execution details (item 9), absence of test review bias (item 10), absence of diagnostic review bias (item 11), absence of clinical review bias (item 12), reporting of uninterpretable/intermediate results (item 13), and withdrawal (item 14). The majority of items are related to bias (items 3, 4, 5, 6, 7, 10, 11, 12 and 14), with only two items relating to variability (items 1 and 2) and three to reporting (items 8, 9 and 13) [18]. For each item, a score of “1” was recorded for “yes” and “0” for “no” or “unknown”. A score of 12 was the cut-off value between high quality and low quality. In addition, in our analysis histopathologic results and/or clinical and imaging follow-up results were used as the “reference standard”, and the examination of PET/MRI was regarded as the “index test”.

### Statistical analysis

Based on the extracted data and bivariate regression models, both per patient and per lesion, we calculated the pooled sensitivity and specificity, which were weighted average estimates using the sample size as the weight for each study. Then, by using the pooled sensitivities and specificities, we also calculated diagnostic odds ratios (DORs), positive likelihood ratios (PLRs) and negative likelihood ratios (NLRs) for PET/MRI. All the pooled data were presented with 95% confidence intervals (CIs). In addition, the summary receiver operating characteristic (SROC) curves were constructed and the area under the curve (AUC) and Q* index were obtained.

An inconsistency index (I^2^) test was performed to assess the degree of heterogeneity between studies, and herein I^2^ describes the percentage of variability in point estimates that is due to heterogeneity rather than sampling error [19]. If the I^2^ value was greater than 50%, meaning that a distinct heterogeneity was observed, the random-effect model was applied; otherwise, the fixed-effect model was used. We investigated the effect of heterogeneity on the diagnostic value of PET/MRI by subgroup analyses based on the study design, quality score, scanning modality and reference standard. We also performed a sensitivity analysis and meta-regression analysis and tested the publication bias and threshold-effect.

All statistical computations were conducted with Stata version 12.0 and Meta-Disc version 1.4. *P* values less than 0.05 were considered to be statistically significant.

## Results

### Eligible studies

**Fig 1:**
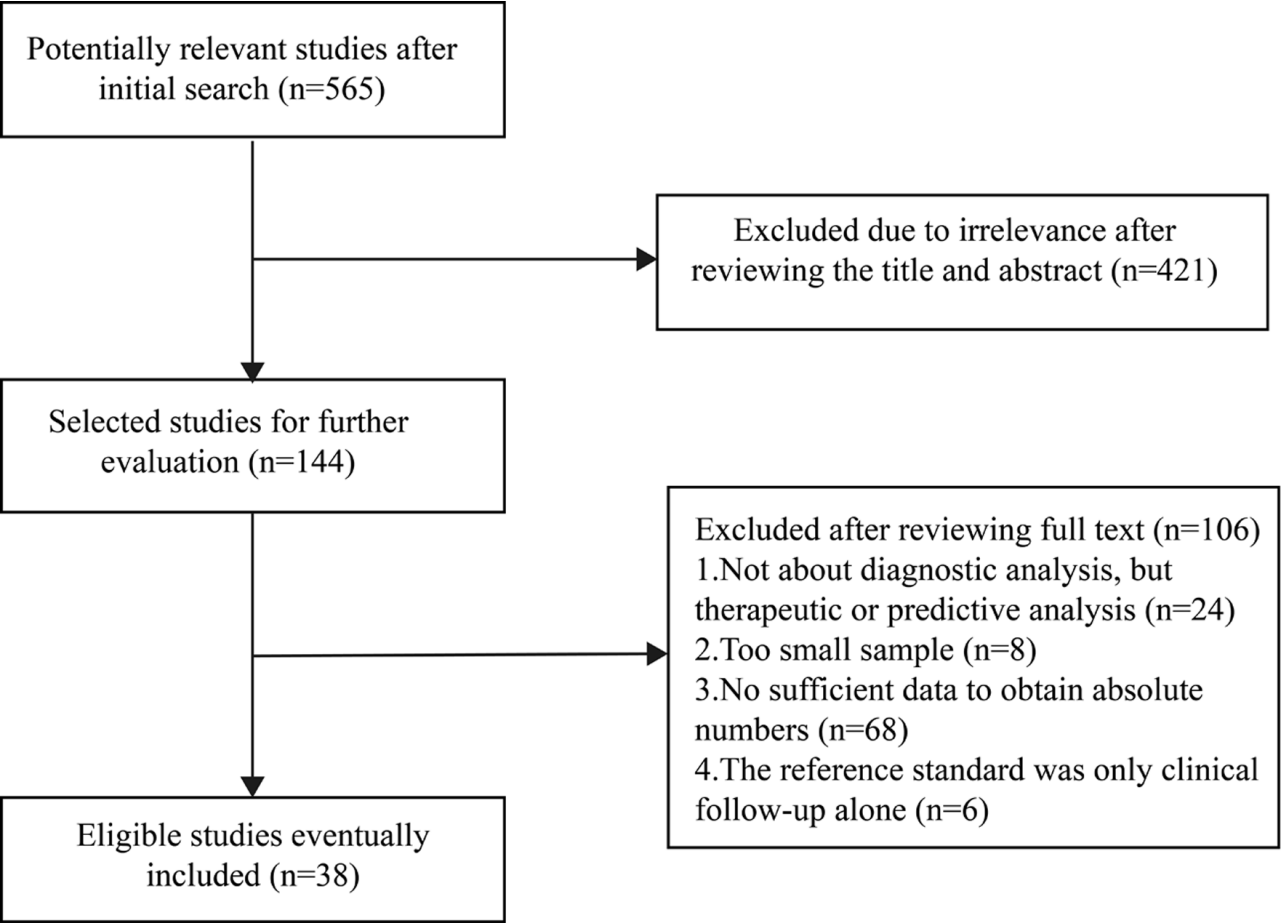
Flow chart showing the process of study selection.

The initial electronic search yielded 565 articles, and after reviewing the title and abstract, 421 were excluded because of irrelevance. The full text of the remaining 144 articles was screened, and 106 studies were excluded for the following reasons: the studies did not provide any diagnostic information beyond therapeutic or predictive value (n = 24); the sample was too small (n = 8); there were insufficient data to obtain absolute numbers of TP, FP, FN or TN results (n = 68); or the reference standard was not histopathologic findings or a combination of histopathology and clinical follow-up (n = 6). Eventually, 38 articles [9–11,13,16,20–52] that included 753 patients and 4234 lesions were included in this meta-analysis. The flowchart of study selection is shown in Fig 1. Of the 38 articles, 17 were retrospective. The combination of histopathologic results and clinical and/or imaging follow-up was regarded as the reference standard in 23 articles. The detailed characteristics of the included studies are shown in Table 1.

**Table 1:** The principal characteristics of included studies.

Study	Year	Origin	Design	Mean age	Gender(M/F)	Cancer type	Blind	Imaging modality	Reference standard	Quality score	Analysis	No. of p/l
**Beiderwellen**	2015	Germany	P	54±13(25–72)	0/19	Cervical cancer, ovarian cancer	B	Integrated PET/MRI	HP+CFU	11	Lesion	78
**Beiderwellen**	2015	Germany	P	57±13	12/20	Malignant melanoma, breast cancer, colorectal cancer and others	ND	Integrated PET/MRI	HP+CFU	9	Lesion	113
**Brendle**	2015	Germany	R	45(10–62)	9/6	Colon cancer, sigmoid cancer, and rectal cancer	B	Integrated PET/MRI	HP+CFU	10	Lesion	180
**Paspulati**	2015	USA	P	59	9/3	Colorectal cancer	B	Sequential PET/MRI	HP+CFU	12	Patient	12
**Grueneisen**	2015	Germany	R	57±13(27–74)	0/24	Ovarian cancer, cervical cancer and endometrial cancer	B	Integrated PET/MRI	HP+CFU	10	Lesion	104
**Nensa**	2015	Germany	P	57.5(20–83)	7/13	metastases, direct infiltration via pulmonary vein, local relapse of primary sarcoma after surgery, Burkitt lymphoma, scar/patch tissue after surgery of primary sarcoma, myxoma, fibroelastoma, CCMA, and thrombus	ND	Integrated PET/MR	HP+CFU	11	Patient	20
**Grueneisen**	2015	Germany	P	48±12(28–73)	0/27	Primary cervical cancer	B	Integrated PET/MRI	HP	12	Patient	27
**Schaarschmidt**	2015	Germany	R	56.5±7.7	23/2	Head and neck squamous cell carcinoma	B	Integrated PET/MRI	HP	13	Lesion	397
**Queiroz**	2015	Swizerland	P	60(37–81)	0/26	Gynaecological malignancies	ND	trimodality PET/CT-MRI	HP+CFU	10	Patient	26
**Grueneisen**	2015	Germany	P	56 ± 12(29–84)	0/49	Primary breast cancer	B	Integrated PET/MRI	HP	12	Lesion	83
**Gatidis**	2015	Germany	P	4.8(1–6)	6/3	Primitive neuroectodermal tumor, posttransplant lymphoproliferative disorder, Rhabdomyosarcoma, Neuroblastoma, Coccygeal teratoma, Neurofibromatosis	ND	Integrated PET/MRI	HP	12	Lesion	29
**Heusch**	2015	Germany	R	59(21–85)	ND	NSCLC, breast carcinoma, cancer of unknown primary site, head and neck tumor, melanoma of the uvea, genitourinary tumor, tumor of the gastrointestinal tract, malignant melanoma, pleural mesothelioma, liver tumor.	Blind	Integrated PET/MRI	HP+CFU/IFU	12	Patient	67
**Taneja**	2014	India	R	50.12(34–75)	0/36	Breast cancer	ND	Integrated PET/MRI	HP+CFU/IFU	10	Patient	26
**Reiner**	2014	Switzerland	P	61(32–79)	33/22	Liver metastasis (colorectal carcinoma, pancreatic carcinoma, gastrointestinal stromal tumour, cholangiocellular carcinoma, gastroesophageal carcinoma)	Blind	Trimodality PET/CT/MRI	HP+CFU	12	Patient/ Lesion	57/120
**Queiroz**	2014	Switzerland	P	63(24–90)	68/19	Head and neck cancer	ND	Trimodality PET/CT/MR	HP+CFU	10	Lesion	117
**Queiroz**	2014	Switzerland	P	63.8(26–86)	53/17	Head and neck cancer	ND	Trimodality PET/CT/MR	HP+CFU	11	Lesion	188
**Platzek**	2014	Germany	P	63(43–82)	30/8	Head and neck cancer	Blind	Ingenuity TF PET/MR	HP	12	Lesion	391
**Platzek**	2014	Germany	R	45(16–74)	16/11	Hodgkin disease, diffuse large B-cell lymphoma, follicular lymphoma, anaplastic large-cell lymphoma, Burkitt lymphoma, cutaneous T-cell lymphoma	Blind	Ingenuity TF PET/MR	HP+CFU	12	Lesion	702
**Loeffelbein**	2014	Germany	R	57(27–72)	21/12	Head and neck cancer	Blind	Software-fused PET+MRI	HP	12	Patient	31
**Kubiessa**	2014	Germany	P	60(42–78)	13/4	Head and neck cancer	Blind	Integrated PET/MR	HP+CFU	12	Lesion	78
**Kitajima**	2014	Japan	R	61.3(38–83)	0/30	Uterine cervical cancer, ovarian cancer, endometrial cancer	Blind	Software-fused PET+MRI	HP+CFU	12	Patient	30
**Kitajima**	2014	Japan	R	57.8(27–88)	0/30	Uterine cervical cancer	Blind	Software-fused PET+MRI	HP+CFU	12	Patient	30
**Heusch**	2014	Germany	P	65(46–86)	11/7	Head and neck squamous cell carcinoma	Blind	Software-fused PET+MRI	HP	12	Lesion	288
**Heusch**	2014	Germany	P	65.1	12/10	NSCLC	Blind	Integrated PET/MR	HP	12	Patient	22
**Grueneisen**	2014	Germany	P	52.8(26–73)	0/48	Cervical cancer, vulvar cancer, vaginal cancer, endometrial cancer, ovarian cancer	Blind	Integrated PET/MRI	HP+CFU	12	Lesion	122
**Bitencourt**	2014	Brazil	P	47.8(29–77)	0/31	Breast cancer	ND	Software-fused PET+MRI	HP	12	Lesion	38
**Vargas**	2013	USA	R	56(28–81)	0/31	Carcinoma of the cervix, endometrium or vagina/vulva	Blind	Software-fused PET+MRI	HP	14	Patient	31
**Nagamachi**	2013	Japan	R	67.1(34–85)	64/55	Pancreatic tumor	Blind	Software-fused PET+MRI	HP	12	Patient	119
**Kohan**	2013	USA	P	66±8	2/9	Lung cancer	Blind	Ingenuity PET/MRI	HP+CFU/IFU	12	Patient	44
**Kitajima**	2013	Japan	R	62.4(30–88)	0/30	Endometrial cancer	Blind	Software-based PET+MRI	HP+CFU/IFU	12	Patient	30
**Kanda**	2013	Japan	R	66.9(37–91)	24/6	Squamous cell carcinoma of the oral cavity or hypopharynx	Blind	Software-based PET+MRI	HP	13	Patient/ Lesion	30/244
**Garibotto**	2013	France	P	51(6–89)	6/9	Neurodegenerative disease, epilepsy, high-grade tumor	ND	Ingenuity TF PET/MR	HP+CFU	10	Patient	15
**Pfluger**	2012	Germany	R	11	77/55	Paediatric oncology	ND	Software-fused PET+MRI	HP+IFU	10	Lesion	813
**Kam**	2010	Australia	R	60(46–75)	15/8	Rectal cancer	ND	Software-fused PET+MRI	HP	12	Patient	23
**Donati**	2010	Switzerland	R	60.2(35–82)	23/14	Hepatic metastases	Blind	Software-fused PET+MRI	HP	13	Patient /Lesion	37/85
**Estrada**	2008	Mexico	P	43	20/10	Primary cerebral tumor	ND	Software-fused PET+MRI	HP+CFU	10	Patient	30
**Huang**	2011	Taiwan	P	54(36–79)	16/1	Buccal squamous cell carcinoma	Blind	Software-fused PET+MRI	HP	13	Lesion	64
**Nakamoto**	2009	Japan	R	62(27–81)	50/15	Head and neck cancer	ND	Software-fused PET+MRI	HP+CFU	9	Patient	46

No: number; ND: unknown or no document; HP: histopathologic results; CFU/IFU: clinical/imaging follow-up; NSCLC: non-small cell lung cancer; P: prospective; R: retrospective; CCMA: caseous calcification of mitral annulus; NSCLC: non-small cell lung cancer; p/l: patient/lesion

### Quality assessment

**Fig 2:**
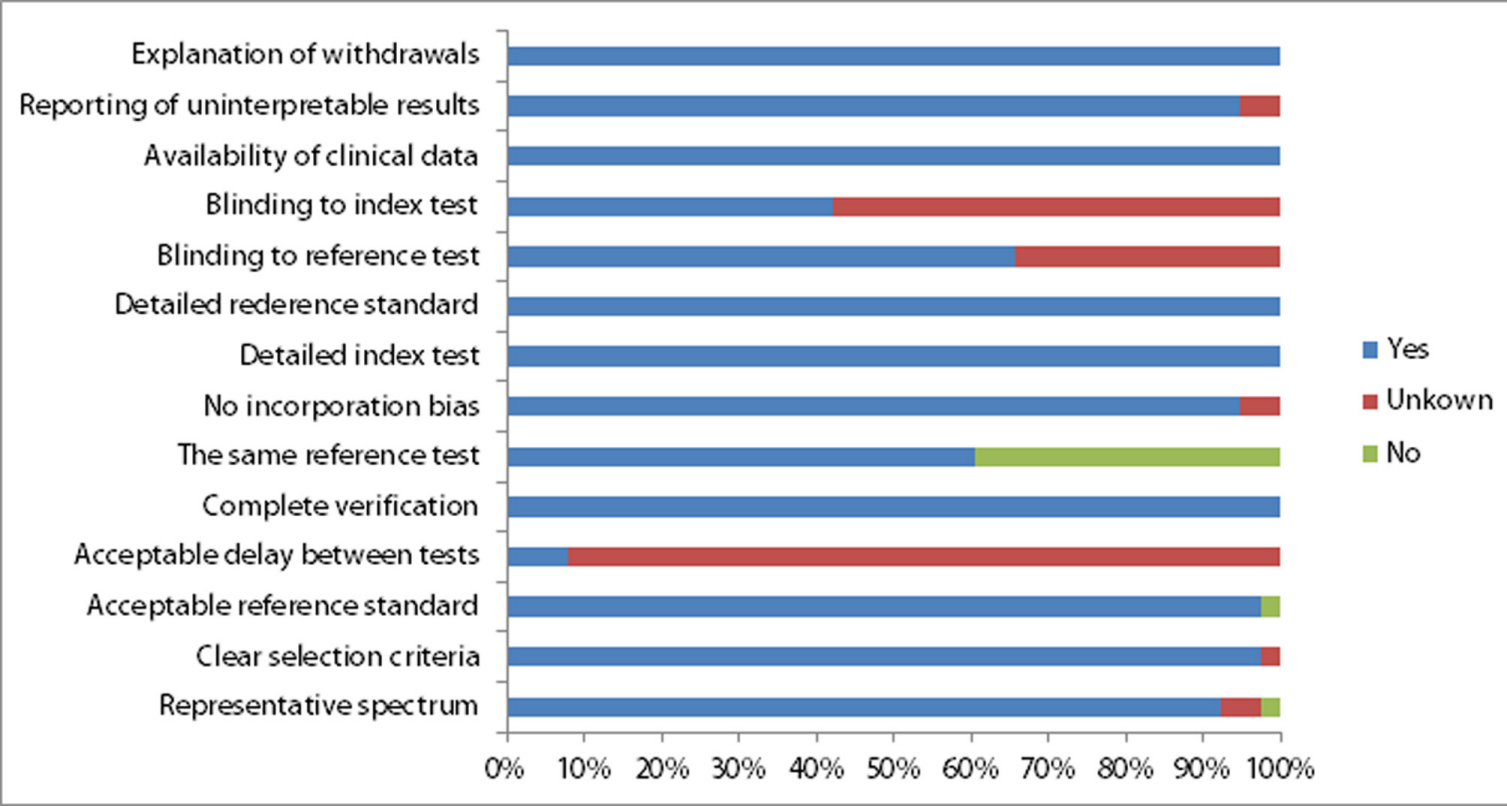
Methodological quality of all eligible studies. Each item is presented as percentages across all included studies.

The results of the QUADAS are shown in Fig 2. Overall, 27 studies (for per-patient and/or per-lesion) fulfilled 12 or more of the 14 items in this meta-analysis, and these were regarded as the studies with high quality. The common weaknesses were centered on whether the time interval between the reference standard and the index test was acceptable (item 4) and whether the reference standard was the same regardless of the index test results (item 6). In addition, 9 studies did not indicate whether the reference standard and index test were masked to one another (item 10 and item 11).

### Diagnostic accuracy of PET/MRI

Per patient, the pooled sensitivity, specificity and DOR for PET/MRI were 0.93 (95% CI, 0.90–0.95), 0.92 (95% CI, 0.89–0.95) and 75.08 (95% CI, 42.10–133.91), respectively. The pooled PLR and NLR estimates were 6.67 (95% CI, 4.83–9.19) and 0.12 (95% CI, 0.07–0.21), respectively. The per-patient sensitivity analysis showed that no individual study contributed to the pooled values whereas, this analysis on a per-lesion level revealed that three studies had significant influence on the overall estimates [29,36,48]. After excluding these three studies, the summarized sensitivity, specificity and DOR per lesion were 0.90 (95% CI, 0.88–0.92), 0.95 (95% CI, 0.94–0.96) and 102.53 (95% CI, 59.74–175.97), respectively. A likelihood ratio syntheses yielded an overall PLR of 10.91 (95% CI, 6.79–17.54) and NLR of 0.13 (95% CI, 0.08–0.19). The results are shown in Table 2.

**Table 2:** The diagnostic accuracy of PET/MRI for detection of malignant lesions.

Study	No. of studies	Sensitivity	I^2^	Specificity	I^2^	AUC	Q*
***Per-patient***							
All	21	0.93(0.90–0.95)	64.8%	0.92(0.89–0.95)	30.4%	0.9545	0.8967
**Study Design**							
Prospective	9	0.94(0.88–0.97)	44.7%	0.90(0.84–0.95)	42.8%	0.9548	0.8971
Retrospective	12	0.92(0.89–0.95)	73.9%	0.93(0.89–0.96)	20.1%	0.9647	0.9115
**QUADAS**							
High quality	15	0.91(0.87–0.94)	65.9%	0.93(0.89–0.96)	34.9%	0.9601	0.9047
Low quality	6	0.98(0.94–1.00)	17.7%	0.89(0.75–0.96)	21.7%	0.9556	0.8982
**Reference standard**							
HP	8	0.93(0.88–0.96)	72.6%	0.93(0.87–0.97)	9.3%	0.9704	0.9205
HP+CFU	13	0.93(0.88–0.96)	61.7%	0.92(0.88–0.95)	42.6%	0.9511	0.8920
**Modality**							
Integrated PET/MRI	10	0.90(0.83–0.94)	54.1%	0.92(0.87–0.96)	33.1%	0.9513	0.8922
Software-Fused PET+MRI	11	0.94(0.91–0.97)	70.6%	0.93(0.88–0.96)	34.3%	0.9691	0.9183
***Per-lesion***							
All	17	0.90(0.88–0.92)	80.0%	0.95(0.94–0.96)	80.0%	0.9641	0.9105
**Study Design**							
Prospective	13	0.91(0.89–0.93)	82.5%	0.93(0.92–0.95)	78.1%	0.9611	0.9062
Retrospective	4	0.85(0.80–0.90)	40.3%	0.97(0.96–0.99)	71.4%	0.9365	0.8731
**QUADAS**							
High quality	12	0.89(0.86–0.92)	83.0%	0.96(0.94–0.97)	77.7%	0.9676	0.9161
Low quality	5	0.91(0.87–0.94)	73.1%	0.91(0.87–0.94)	81.9%	0.9496	0.8899
**Reference standard**							
HP	9	0.86(0.82–0.90)	80.2%	0.96(0.94–0.97)	82.7%	0.9635	0.9097
HP+CFU	8	0.92(0.89–0.94)	78.9%	0.92(0.89–0.94)	73.1%	0.9683	0.9170
**Modality**							
Integrated PET/MRI	12	0.92(0.89–0.94)	71.3%	0.95(0.93–0.96)	83.2%	0.9652	0.9122
Software-Fused PET+MRI	5	0.83(0.77–0.88)	87.5%	0.95(0.93–0.97)	72.3%	0.9610	0.9059

AUC: area under the curve, HP: histopathologic results, CFU: clinical follow-up; in parentheses: the 95% confident interval

**Fig 3:**
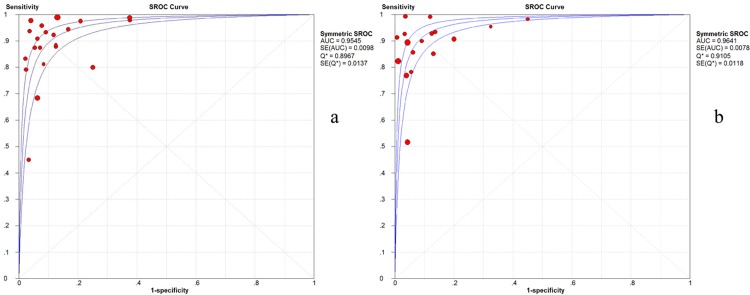
SROC curves for PET/MRI on a per-patient level (a) and a per-lesion level (b). Each solid circle represents a study in this meta-analysis.

The SROC curves and the Q* index on both a per-patient basis and a per-lesion basis are presented in Fig 3.

### Heterogeneity analysis

The I^2^ estimates were 64.8% for pooled sensitivity on a per-patient basis, 80.0% for pooled sensitivity on a per-lesion basis, and 80.0% for pooled specificity on a per-lesion basis, indicating significant heterogeneity among included studies. The heterogeneity of pooled specificity was low only on a per patient basis (I^2^ = 30.4%). The results of the heterogeneity analysis are shown in Table 2

**Fig 4:**
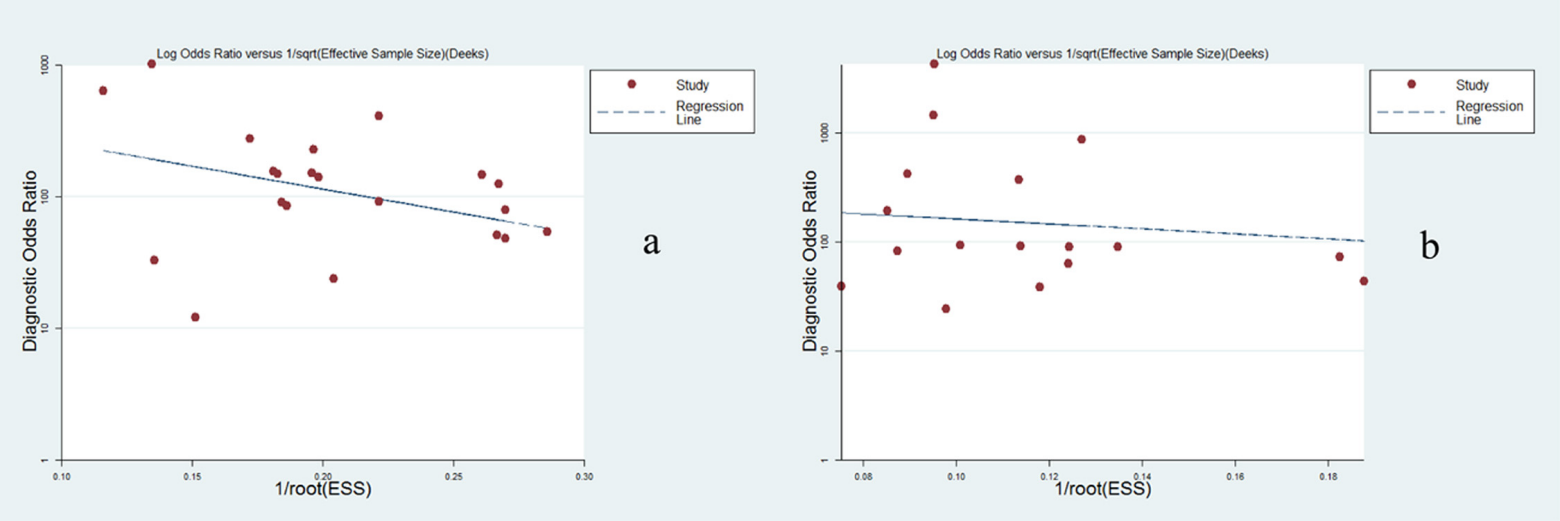
Deeks funnel plot of asymmetry test for publication bias on a per-patient level (a) and a per-lesion level (b). The nonsignificant slope indicates the absence of publication bias.

To explore the source of heterogeneity, Spearman’s correlation test was used to evaluate the threshold effect, and its coefficient was determined to be 0.527 (*P* = 0.014) per patient and 0.358 (*P* = 0.16) per lesion, indicating an absence of the threshold effect. With regard to publication bias, the results of the Deeks funnel plots were not significant (*P* = 0.185 for per patient, *P* = 0.748 for per lesion), suggesting no major publication bias (Fig 4).

The results of the subgroup analysis are also shown in Table 2. The study design, quality score, reference standard and scanning modality did not significantly influence the reported sensitivities and specificities of PET/MRI on a per-lesion level (*P* > 0.05). On a per-patient level, only scanning modality had a significant influence on the sensitivity of PET/MRI. In other words, the subgroup of integrated PET/MRI had a higher sensitivity than that of software-fused PET/MRI (*P* < 0.05). The results of a single-factor meta-regression analysis showed that no factor significantly influenced the diagnostic accuracy of PET/MRI.

With regard to cancer type, due to limited information and small sample sizes, we only pooled the diagnostic estimates for head and neck cancer on a per-lesion level. The corresponding estimates of 8 studies [9,16,20,34,35,39,46,49] were 0.83 (95% CI, 0.79–0.87) for sensitivity, 0.96 (95% CI, 0.94–0.97) for specificity and 81.20 (95% CI, 42.27–155.97) for DOR.

## Discussion

When making decisions regarding cancer therapy, it is necessary to have precise knowledge of the local tumor stage as well as to detect potential metastasis to regional lymph nodes and distant organs. In this study, we performed a meta-analysis to evaluate the diagnostic accuracy of integrated PET/MRI or software-fused PET/MRI for tumor staging in patients with various cancers, and the results showed that PET/MRI had excellent diagnostic performance for the overall detection of malignancies.

Likelihood ratios enable characterizing clinical diagnostic tests to establish diagnoses for individual patients [53]. The discriminating ability is better, with a higher PLR and lower NLR. In general, a PLR greater than 10.0 would be required to confirm the presence of disease, and an NLR less than 0.1 would be required to eliminate the possibility. Per patient, the PLR was not high enough to diagnose malignancies, but it was high enough per lesion. Because the NLRs were not low enough on a per-patient or a per-lesion level, a negative PET/MRI finding alone might not rule out a malignancy. The DOR is the ratio of PLR to NLR and ranges from 0 to infinity. The greater the DOR, the better the performance of the diagnostic test, and the DOR in this meta-analysis showed that PET/MRI performed well.

A comparative study of PET/MRI, PET/CT, MRI, and CT imaging for assessing surrounding tissue invasion of advanced buccal squamous cell carcinoma reported that PET/MRI had highest sensitivity and specificity among the 4 modalities (90.0%/90.9%, 80.0%/84.1%, 80.0%/79.5%, and 55.0%/81.8%, respectively) [37]. Similarly, in head and neck cancer, PET-MRI fusion has higher sensitivity/specificity for tumor staging (89%/100%), compared with that of MRI (79%/66%) and that of PET (82%/100%) [49]. In addition, Kitajima found that for T-status staging of cervical cancer, PET/MRI proved significantly more accurate than PET/CT (83.3% vs. 53.3%).[10] Summarizing the available evidence, in several specific regions such as head, neck and pelvic regions, PET/MRI might be more accurate for primary tumor staging compared with conventional imaging methods or PET/CT. One potential explanation might be the high soft-tissue contrast of MRI in these regions [10,37,38]. MRI could provide an accurate assessment of the local tumor extent to determine the extent of tumor resection and the adjuvant therapy. However, due to scar tissue, loss of symmetry and side shift, MRI alone may not help differentiate between non-neoplastic and neoplastic changes in operated regions [54]. Currently, PET might provide additional metabolic information for the differential diagnosis, although it might have some false positive findings [55]. Therefore, the detection of invasion of the adjacent anatomical structures is a potential advantage of PET/MRI for primary tumor staging. However, in some other common tumors, such as NSCLC and liver metastatic lesions, PET/MRI does not provide higher accuracy for detecting malignancies [33,40]. With regard to tumor N staging, several previous studies have shown that PET/MRI and PET/CT were of equal diagnostic accuracy. Kohan reported that PET/MRI using three-segment model attenuation correction showed substantial inter-observer agreement and a similar performance to PET/CT in N staging of lung cancer. In head and neck cancers, PET/MRI did not significantly improve the accuracy for detecting cervical lymph node metastases compared to MRI or PET [35]. In addition, PET/MRI is feasible for lymphoma staging and has a high sensitivity and specificity for nodal involvement in lymphoma (93.8% for sensitivity and 99.4% for specificity) [36]. Because the detection of lymph node metastases is predominantly based on the functional information of PET, which is highly sensitive, the differences between PET/MRI and PET/CT were not significant. In a few studies, additional functional MRI sequences, such as DWI, were shown to potentially help detect metastatic lymph nodes showing no enlargement [56]. However, several other studies have shown no additional diagnostic value of DWI in PET/MRI for the detection of metastases [39,41,57]. For M staging, only one study was analyzed, and the result suggested that no significant differences were observed between PET/MRI and PET/CT [24]. By increasing the sample size and testing efficiency and reducing random error, our meta-analysis revealed that PET/MRI has excellent diagnostic accuracy for tumor staging on both a per-patient and a per-lesion level. However, the heterogeneity between studies was significant, and the sources should be investigated.

To determine whether the threshold effect was an impact factor, it was evaluated by Spearman’s correlation. The estimate of 0.358 (*P* = 0.16) per lesion revealed that no significant threshold effect existed, while the estimate of 0.527 (*P* = 0.014) per patient indicated a notable threshold effect. The reason for the notable threshold effect lies in the combination of high sensitivity and low specificity or vice versa. For example, Nakamoto et al showed the highest sensitivity (1.00) and the lowest specificity (0.67) [13]. By contrast, Kam showed the lowest sensitivity (0.44) and the highest specificity (1.00) [50]. The feature of significant differences was mainly caused by different diagnostic cut-off values in individual studies. Additionally, the results of the Deeks funnel plot asymmetry test provided no evidence of notable publication bias (*P* = 0.185 per patient, *P* = 0.748 per lesion). Based on the putative factors of study design, quality score, reference standard and scanning modality, we performed the subgroup analysis, and the results showed that only scanning modality significantly contributed to the heterogeneity on a per-lesion level. The integrated PET/MRI system can achieve better and more consistent image registration accuracy than can the software-based fusion of PET and MRI, further yielding better diagnostic performance [58].

We performed an additional assessment for other sources of heterogeneity. The diagnostic performance for staging might depend on primary tumor type, target organ of potential metastases and scanning protocols. For example, while PET/MRI may be superior for hepatic staging or head and neck cancers, its application can be limited for detecting small pulmonary metastases [59]. However, we could not perform any further analysis for each cancer type due to small sample sizes and limited information. Regarding the scanning protocol, for which there is no consensus, most studies used FDG PET/unenhanced T1-weighted MRI images, but some studies [10,11,38] used the contrast enhanced imaging or organ-specific imaging protocol. Moreover, some studies investigated the value of software-fused PET/MRI while others focused on the value of integrated PET/MRI; the latter had more precise anatomic fusion and location. Finally, the sequence of subsequent PET/MRI acquisitions might influence the diagnostic results [16]. The delayed PET acquired during FDG PET/MRI would show more lesions because of tracer accumulation in malignant lesions [60]. All these differences may have biased the pooled estimates of PET/MRI for tumor staging.

There are some limitations in this study. First, the exclusion of conference abstracts and letters may have led to reporting bias. Although the Deeks test revealed an absence of publication bias, it could still exist because studies with optimistic results might be published more easily. Second, we did not perform subgroup analyses for each cancer type due to limited information and small sample sizes. Third, there was no standard scanning modality and protocol, which might affect the pooled accuracy of PET/MRI and be a main source of between-studies heterogeneity. The reference standards used were not the same and included histopathological findings alone and in combination with clinical/imaging follow-up. Fourth, approximately 55% of the included studies were retrospective, which might be a potential limitation because of the risk that investigators might have known the results of other imaging modalities or clinical examinations before evaluating the PET/MRI images. Fifth, we did not compare the value of PET/MRI with that of PET/CT due to limited information, which might lead to an incomplete knowledge of PET/MRI in clinical practice.

## Conclusions

Current evidence shows that PET/MRI has excellent diagnostic performance for overall tumor staging. Large, multicenter and prospective studies with standard scanning protocols are required to evaluate the diagnostic value of PET/MRI for individual cancer types. Additionally, a comparison of PET/MRI with other modalities especially PET/CT is urgently needed.

## Supporting Information

S1 PRISMA Checklist PRISMA 2009 Checklist. (DOC)
